# The effects of stillbirth and abortion on the next pregnancy: a longitudinal study

**DOI:** 10.1186/s12905-021-01485-0

**Published:** 2021-09-25

**Authors:** Berhanu Elfu Feleke, Teferi Elfu Feleke, Azezu Asres Nigussie, Eyaya Misgan

**Affiliations:** 1 Department of Epidemiology and Biostatistics, University of Bahir Dar, Bahir Dar, Ethiopia; 2 Department of Pediatrics, Wolkite University, Wolkite, Ethiopia; 3 Department of Midwifery, University of Bahir Dar, Bahir Dar, Ethiopia; 4 Department of Gynecology and Obstetrics, University of Bahir Dar, Bahir Dar, Ethiopia

**Keywords:** Fetal death, Pregnancy outcome, Stillbirth, Abortion, Ethiopia

## Abstract

**Background:**

Abortion and stillbirths are the common obstetrics condition in Ethiopia and their effect on the next pregnancy was not well identified in resource limited settings. The aim of the study was to assess the effect of stillbirth and abortion on the next pregnancy.

**Methods:**

A prospective cohort study design was implemented. The study was conducted in Mecha demographic surveillance and field research center catchment areas. The data were collected from January 2015 to March 2019. Epi-info software was used to calculate the sample size. The systematic random sampling technique was used to select stillbirth and abortion women. Poison regression was used to identify the predictors of MCH service utilization; descriptive statistics were used to identify the prevalence of blood borne pathogens. The Kaplan Meier survival curve was used to estimate survival to pregnancy and pregnancy related medical disorders.

**Results:**

1091 stillbirth and 3,026 abortion women were followed. Hepatitis B was present in 6% of abortion and 3.2% of stillbirth women. Hepatitis C was diagnosed in 4.7% of abortion and 0.3% of stillbirth women. HIV was detected in 3% of abortion and 0.8% of stillbirth women. MCH service utilization was determined by knowledge of contraceptives [IRR 1.29, 95% CI 1.18–1.42], tertiary education [IRR 4.29, 95% CI 3.72–4.96], secondary education. [IRR 3.14, 95% CI 2.73–3.61], married women [IRR 2.08, 95% CI 1.84–2.34], family size [IRR 0.67, 95% CI 1.001–1.01], the median time of pregnancy after stillbirth and abortion were 12 months. Ante-partum hemorrhage was observed in 23.1% of pregnant mothers with a past history of abortion cases and post-partum hemorrhage was observed in 25.6% of pregnant mothers with a past history of abortion. PREGNANCY INDUCED DIABETES MELLITUS was observed 14.3% of pregnant mothers with a past history of stillbirth and pregnancy-induced hypertension were observed in 9.2% of mothers with a past history of stillbirth.

**Conclusion:**

Obstetric hemorrhage was the common complications of abortion women while Pregnancy-induced diabetic Mellitus and pregnancy-induced hypertension were the most common complications of stillbirth for the next pregnancy.

## Background

Delivery of the fetus with no sign of life after 28 weeks of gestation was labeled as stillbirth [1]. According to the world health organization report, more than 2.6 million stillbirths were reported annually [2]. More than 75% of stillbirths occur in sub-Saharan Africa and South Asia [3]. Placental problems, childbirth complications, infections, congenital anomalies, and intrauterine growth retardation were some of the causes for stillbirth [4, 5].

Expulsion of the product of pregnancy before viability, usually 28 weeks of gestational age defines abortion in resource limited settings [6]. Globally more than 25 million unsafe abortions women were reported in 2014, and most of them occur in developing countries [7]. Abnormal chromosomes, maternal medical disorders, and unhealthy lifestyles are some of the reasons for abortion women [8]. Uterine perforation, infections, bowel and bladder injury, excessive bleeding are some of the complications of abortion women [9, 10].

Abortion was among the top leading causes of maternal mortality. According to the Ethiopian demographic and health surveillance 2016 report, the maternal mortality ratio of Ethiopia was 412 per 100,000 live births [11]. Ethiopia tries to reduce the complications of abortion by expanding the family planning services accessibility and by drafting the legal abortion law [12].

Availing the maternal and child health services significantly decrease maternal mortality. Report suggests that MCH service utilization was determined by age, birth rank, educational level, monthly income, number of children, occupation, knowledge about sexually transmitted infections, educational status, residence, parity, partner communication, and the presence of TV/radio [13–19].

In resource limited settings, evidence on the effect of stillbirth and abortion women on the next pregnancy was scarce, which affects the quality of maternal and child health services. This research work generates evidence and improves the quality and utilization of maternal and child health services.

The objectives of this study were: to estimate the MCH service utilization after stillbirth and abortion, to assess the prevalence of blood borne pathogens among stillbirth and abortion women, to estimate the median time of pregnancy after stillbirth and abortion women, to estimate the risk of obstetric hemorrhage and pregnancy-induced medical disorders after stillbirth and abortion women.

## Methods

A Prospective cohort study design was implemented. The study was conducted in the Mecha demographic surveillance and field research center catchment areas, Mecha district. The district was located 30 km away from Bahir Dar, the capital city of Amhara National Regional State. The data were collected from January 2015 to March 2019. The target population for the study was all mothers with stillbirth or abortion. Women out migrated from the catchment areas were excluded. During update data collection, pregnant mothers with chronic diabetes mellitus or chronic hypertension were excluded. The sample size was calculated using Epi-info software version 7 with the assumption of 95% CI, power of 90, 3:1 ratio of abortion women to stillbirth women, a risk ratio of 1.12 and 10% non-response rate; give 1091 stillbirth and 3171 abortion women. The systematic random sampling technique was used to select both the stillbirths and abortion women. They were recruited after pregnancy termination. The study participants were selected from the government health facilities of the Mecha district. Interviewing the patient was conducted by midwife professionals and laboratory samples were collected by laboratory technologists. Baseline data were collected from the post-abortion women and stillbirth women in the gynecologic ward using the patient interviews. Then, every three months update data were collected from each study participant from their residence using Mecha demographic surveillance and field research center frame. Clinician-Administered post-traumatic stress disorder Scale (PTSD) was used to screen for post-traumatic stress disorder. Laboratory samples were collected by laboratory technologists. For each study participant, 5 ml (ML) venous blood was collected using an aseptic technique and an Enzyme-linked immune Sorbent assay (ELIZA) test was performed to screen for the presence of hepatitis B and hepatitis C. The standard operating procedures (SOP) were followed. For pregnant mothers, Peripheral blood was collected to measure blood glucose levels. American diabetic association criteria was used to diagnose gestational diabetic mellitus [20]. Focused ante-natal care (FANC) was given for all pregnant mothers to the nearby health facility.

To increase the quality of data; pretest was performed, the data collection was closely supervised, SOP was adhered to all laboratory procedures, and training was given for all data collectors and supervisors.

Data were entered into the computer using EPI-info software and transferred to SPSS for analysis. Descriptive statistics were used to present the profile of study participants, MCH service utilization and estimate pregnancy-related complications after stillbirth and abortion women. Poisson regression was used to identify the determinants of MCH service utilization after stillbirth and abortion women. The Kaplan Meier survival curve was used to estimate time to pregnancy and pregnancy-related complications.

## Results

A total of 4117 women was included giving for the response rate of 96.6%, 64 women were out-migrated from the study areas and the medical records of 81 women were incomplete. Stillbirth constitutes 1042 study participants. The proportions of stillbirth and abortion women completed the survey was 95.5% and 96.9% respectively. The mean age of the study participant was 21.92 years [SD (standard deviation ± 4.9 years)]. (Table 1).

**Table 1:** Profile of the study participants (n = 4117)

SN^a^	Variables		Stillbirth		Abortion		*P* value
			Frequency	%	Frequency	%	
1.	Knowledge of contraceptive	Present	955	91.7	456	14.8	< 0.01
		Absent	87	8.3	2619	85.2	
2.	Resident	Rural	1020	97.9	2210	71.9	< 0.01
		Urban	22	2.1	865	28.1	
3.	Educational status	Illiterate	66	6.3	603	19.6	< 0.01
		Informal	76	7.3	1147	37.6	
		Elementary	2	0.2	117	5.8	
		Secondary	36	3.5	1009	32.8	
		Tertiary	862	82.7	139	4.5	
4.	TV/radio	Present	367	35.2	260	8.5	< 0.01
		Absent	675	64.8	2815	91.5	
5.	Marital status	Single	46	4.4	2803	91.2	< 0.01
		Married	981	94.1	272	8.6	
		Divorced	11	1.1	0	0	
		Widowed	4	0.4	0	0	
6.	Family size	≤ 4	740	71	1365	44.5	< 0.01
		> 4	302	29	1710	55.6	
7.	Hepatitis C	Positive	3	0.3	144	4.7	< 0.01
		Negative	1039	99.7	2931	95.3	
8.	HIV	Positive	8	0.8	91	3	< 0.01
		Negative	1034	99.2	2984	97	
9.	Hepatitis B	Positive	33	3.2	185	6	< 0.01
		Negative	1009	96.8	2890	94	
10.	Partner communication about MCH	Present	350	33.6	1090	35.4	0.27
		Absent	692	66.4	1085	64.5	

^a^Serial number

From 3075 abortion women, 24.09% of study participants mention rape as a reason for abortion women and 58.08% of study participants not volunteer to mention the reason for abortion women. Post traumatic stress syndrome (PTSD) was observed in 44.9% of study participants (Table 2).

**Table 2:** Post traumatic stress disorder in the post abortion and stillbirth women (n = 4117)

	Absent		Mild		Moderate		Severe		Extreme	
	Frequency	%	Frequency	%	Frequency	%	Frequency	%	Frequency	%
Stillbirth	324	31.1	79	7.6	420	40.3	174	16.7	45	4.3
Abortion	1944	63.2	1131	36.8	0	0	0	0	0	0

During the consecutive 3 years, maternal and child health service (MCH) was utilized by 42.1% of study participants. After adjusting for residence, marital status, stillbirth, abortion, family size, age, gravidity, the presence of TV/Radio, knowledge on contraceptive, educational status, access to contraceptives; MCH service utilization was associated with age, knowledge of contraceptives, educational status, the presence of TV/Radio, marital status, and family size (Table 3).

**Table 3:** Poisson regression output for determinants for MCH services utilization (n = 4117)

Variables	B	Standard error	*P* value	IRR	95% CI for IRR	
					Lower	Upper
Knowledge of contraceptives	.257	.0482	.000	1.293	1.176	1.421
Access to contraceptives	.088	.0387	.023	1.092	1.012	1.178
Tertiary education	1.458	.0729	.000	4.296	3.724	4.955
Secondary education	1.143	.0710	.000	3.137	2.729	3.605
Primary education	− .822	.2041	.000	.440	.295	.656
Informal education	− 1.097	.1026	.000	.334	.273	.408
Presence of TV/Radio	.085	.0317	.007	1.089	1.023	1.159
Widowed	.437	.2606	.093	1.548	.929	2.580
Divorced	.657	.1599	.000	1.929	1.410	2.639
Married	.730	.0612	.000	2.075	1.841	2.340
High family size	− .405	.0325	.000	.667	.626	.711
Age	.005	.0023	.027	1.005	1.001	1.010

*IRR* incident rate ratio

**Fig. 1:**
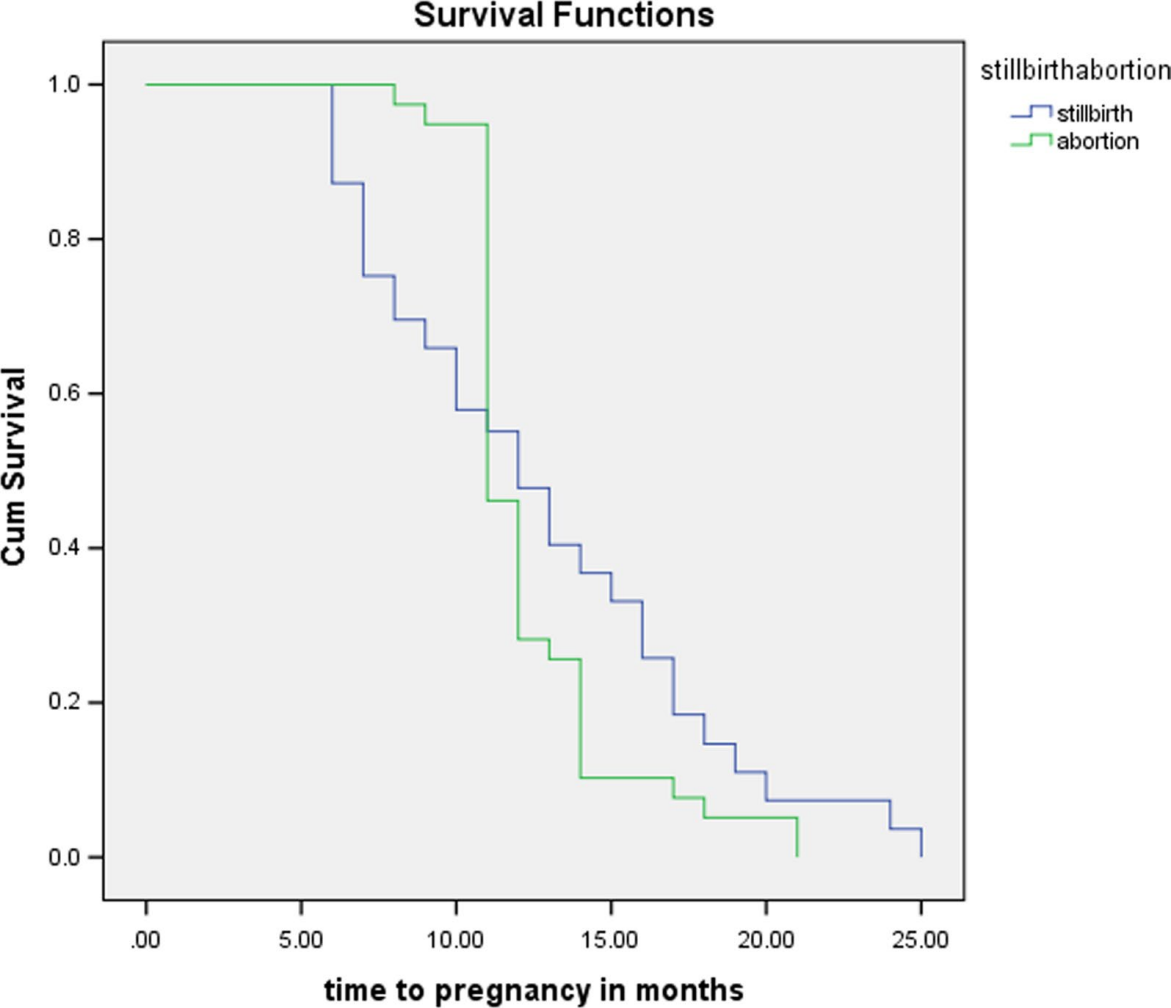
Time to pregnancy after stillbirth and abortion

In the subsequent 3 years, 22.8% of study participants become pregnant and the median time of pregnancy was 12 months (Fig. 1).

**Fig. 2:**
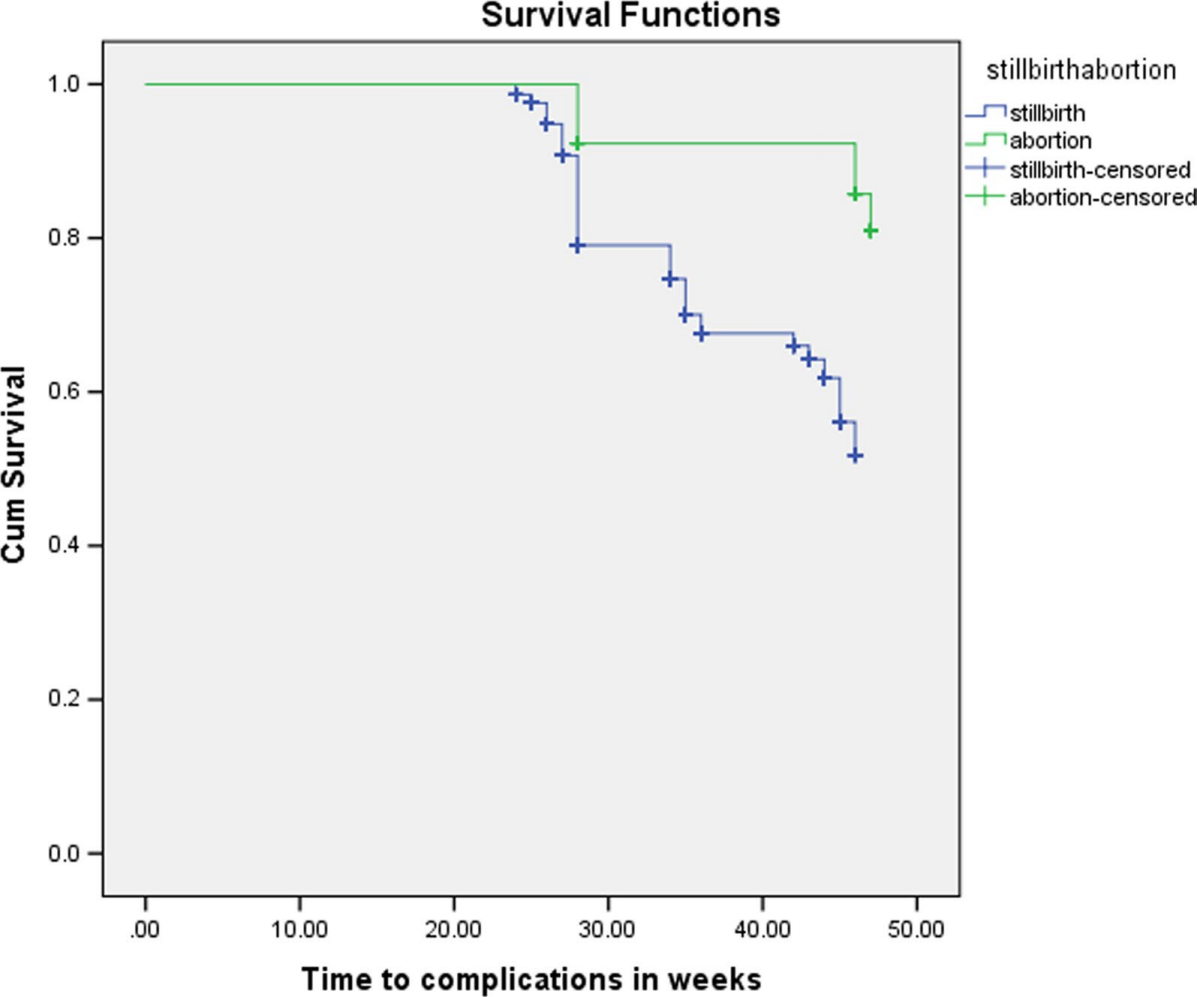
Kaplan Meier survival curve for the pregnancy-related complications

Obstetric hemorrhage was the main complication of abortion for the next pregnancy, antepartum hemorrhage (APH) was observed in 23.1% of pregnant mothers with a past history of abortion and post-partum hemorrhage (PPH) was observed in 25.6% pregnant mothers with past history of abortion (Fig. 2).

Gestational diabetes mellitus and pregnancy-induced hypertension were the common complications of stillbirth for the next pregnancy; PREGNANCY INDUCED DIABETES MELLITUS was observed 14.3% of pregnant mothers with a past history of stillbirth and pregnancy-induced hypertension were observed on 9.2% of mothers with a past history of stillbirth (Table 4).

**Table 4:** Complications of stillbirth and abortion for the next pregnancy (n = 939)

	APH		PPH		Pregnancy induced diabetes mellitus		Pregnancy induced hypertension	
	Frequency	%	Frequency	%	Frequency	%	Frequency	%
Stillbirth	109	12.1	109	12.1	129	14.3	83	9.2
Abortion	9	23.1	10	25.6	4	10.3	2	5.1

*APH* ante-partum haemorrhage, *PPH* post-partum hemorrhage

## Discussion

Totally, 1042 stillbirths and 3026 abortion women were included. Most of the abortion women were single by marital status (91.2%) but only 4.4% of stillbirth women were single. This finding agrees with researcher results from Nepal [21]. This occurs to the reason that pregnancy before marriage is usually unintended and unsupported ending with an abortion [22].

58.1% of abortion women do not want to report the reason for abortion, but, post-traumatic stress disorder was present in 36.8% of the post-abortion women and 68.9% of the stillbirth women. This finding was in line with the 2017 finding [23]. The low prevalence of PTSD among post-abortion women indicates the high proportion of unintended pregnancy in the group [24].

The prevalence of hepatitis among abortion women was two folds higher than stillbirth women. Hepatitis B was present on 6% of the abortion women and 3.2% of the stillbirth women. This figure was higher as compared to the 2016 finding from Ethiopia [25]. This indicates that risky sexual behavior is increasing from time to time.

HIV was detected in 3% of abortion and 0.8% of stillbirth women. This finding was in line with the Uganda research outputs [26]. This is due to the reason that most abortion women were single with their marital status; they might have multiple sexual partners, which finally increase the risk of acquiring HIV.

Hepatitis C was diagnosed in 4.7% of abortion and 0.3% of stillbirth women. This finding agrees with the research results from Egypt [27].

In the consecutive 3 years, MCH service was utilized by 75.7% of stillbirth women, but only 5.1% of abortion women utilized MCH service. The median time of pregnancy was 12 months for stillbirth and 11 months for abortion. This finding was in line with finding from Zambia [28]. This indicates that the tendency for repeated abortion was higher.

MCH service utilization was 29% higher in the presence of good knowledge regarding contraceptives [IRR 1.29, 95% CI 1.18–1.42]. The Ghanaian research article also reported poor knowledge of contraceptives among abortion women [29]. This finding indicates that women should get access to family planning intervention to reduce the risk of unintended pregnancy.

MCH service utilization was 4.3 folds higher among women with tertiary education [IRR 4.29, 95% CI 3.72–4.96], 3.14 folds higher among women in secondary education. [IRR 3.14, 95% CI 2.73–3.61], however, primary education was not helping women to use the MCH services. The previous finding from Ethiopia report the same results [30]. This indicates the government should extend its directions from primary education to secondary and above.

MCH service utilization was two folds higher among married women [IRR 2.08, 95% CI: 1.84 -2.34]. This finding was in line with the Nigeria research article [13]. This is due to the reason that the probability of partner communication regarding MCH was high among married women than single women. Additionally, married women to have access to MCH services related to plan and helped pregnancy.

MCH service utilization was 49% lower in women living with high family-sized households [IRR 0.67, 95% CI 1.001– 1.01]. This finding was in agreement with findings from Sub-Saharan African countries [31]. This indicates that people living in overcrowded areas will not have awareness about MCH services.

Antepartum hemorrhage (APH) was observed in 23.1% of pregnant mothers with a past abortion women and post-partum hemorrhage (PPH) was observed in 25.6% pregnant mothers with a past history of abortion women. Obstetric hemorrhage was a common complication of abortion for the next pregnancy. This is because the uterine procedures performed during abortion affects the integrity of the uterine wall predisposing risk factors like placenta previa and abruption [32, 33].

Stillbirth complicate the next pregnancy by predisposing the women for pregnancy induced diabetes mellitus and pregnancy induced hypertension; pregnancy induced diabetes mellitus was observed in 14.3% of pregnant mothers with a past history of stillbirth and pregnancy-induced hypertension was observed in 9.2% of mothers with a past history of stillbirth. This finding agrees with the previous finding from the same study area [34]. This is due to the endocrine effects of abortion and stillbirth [35, 36].

This research work did not assess the effects of stillbirth or abortion on the child health, and this is the limitation of this research.

## Conclusion

Abortion women had poor knowledge of contraceptives, and unintended pregnancy was the predominant cause of abortion. Blood borne pathogens were common among abortion women. Primary education was not helpful to utilize MCH services. Obstetric hemorrhage was the common complications of abortion women for the next pregnancy. Pregnancy-induced diabetic Mellitus and pregnancy-induced hypertension were the most common complications of stillbirth for the next pregnancy.

## Recommendations

The family planning program should extend its intervention to high school students. The post-abortion care should incorporate intervention for the blood borne pathogens. Emphasis should be given for women to accomplish at least secondary education. Pregnant women with the previous history of abortion should closely be followed to avert the risk of obstetric hemorrhage.

## Data Availability

All data generated or analyzed during this study are included in this published article.

## Abbreviations

APH: antepartum hemorrhage
B: beta-coefficient
CI: confidence interval
ELIZA: enzyme-linked immune Sorbent assay
FANC: ante-natal care
HIV: human immunodeficiency virus
IRR: incident rate ration
MCH: maternal and child health
ML: milliliter
PPH: post-partum hemorrhage
PTSD: post-traumatic stress disorder scale
SD: standard deviation
SN: serial number
SOP: standard operating procedures
